# Altered glucocorticoid metabolism represents a feature of macroph‐aging

**DOI:** 10.1111/acel.13156

**Published:** 2020-05-28

**Authors:** Jenny Vanessa Valbuena Perez, Rebecca Linnenberger, Anna Dembek, Stefano Bruscoli, Carlo Riccardi, Marcel H. Schulz, Markus R. Meyer, Alexandra K. Kiemer, Jessica Hoppstädter

**Affiliations:** ^1^ Pharmaceutical Biology Department of Pharmacy Saarland University Saarbrücken Germany; ^2^ Pharmacology Department of Medicine Perugia University Perugia Italy; ^3^ Institute for Cardiovascular Regeneration Goethe University Frankfurt am Main Germany; ^4^ German Center for Cardiovascular Research (DZHK) Partner Site RheinMain Frankfurt am Main Germany; ^5^ Department of Experimental and Clinical Toxicology Institute of Experimental and Clinical Pharmacology and Toxicology Center for Molecular Signaling (PZMS) Saarland University Homburg Germany

**Keywords:** cellular immunology, cytokines, inflammation, mononuclear cell, mouse models, reactive oxygen species, steroid control of aging, TSC22D3

## Abstract

The aging process is characterized by a chronic, low‐grade inflammatory state, termed “inflammaging.” It has been suggested that macrophage activation plays a key role in the induction and maintenance of this state. In the present study, we aimed to elucidate the mechanisms responsible for aging‐associated changes in the myeloid compartment of mice. The aging phenotype, characterized by elevated cytokine production, was associated with a dysfunction of the hypothalamic–pituitary–adrenal (HPA) axis and diminished serum corticosteroid levels. In particular, the concentration of corticosterone, the major active glucocorticoid in rodents, was decreased. This could be explained by an impaired expression and activity of 11β‐hydroxysteroid dehydrogenase type 1 (11β‐HSD1), an enzyme that determines the extent of cellular glucocorticoid responses by reducing the corticosteroids cortisone/11‐dehydrocorticosterone to their active forms cortisol/corticosterone, in aged macrophages and peripheral leukocytes. These changes were accompanied by a downregulation of the glucocorticoid receptor target gene glucocorticoid‐induced leucine zipper (GILZ) in vitro and in vivo. Since GILZ plays a central role in macrophage activation, we hypothesized that the loss of GILZ contributed to the process of macroph‐aging. The phenotype of macrophages from aged mice was indeed mimicked in young GILZ knockout mice. In summary, the current study provides insight into the role of glucocorticoid metabolism and GILZ regulation during aging.

## Graphical abstract

The present study examined the age‐associated changes in glucocorticoid metabolism in macrophages in order to improve the current understanding of “inflammaging.”

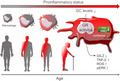

## 1 INTRODUCTION

While the adaptive immune system usually deteriorates with age, innate immune cells can cause a chronic, low‐grade systemic inflammatory state, termed “inflammaging” (Franceschi et al., 2000). This chronic inflammation, fueled by continuous exposure to exogenous and endogenous stimuli during the lifespan, is considered by geroscience as one of the seven pillars of aging, and largely determines the onset of age‐related diseases (Franceschi, Garagnani, Parini, Giuliani, & Santoro, 2018). Different factors, such as cellular senescence, altered metabolic activity, and endocrine disorders contribute to sustaining inflammaging (Bandaranayake & Shaw, 2016).

The macrophage is a key driver of age‐related inflammation. The theory of inflammaging has been intertwined with “macroph‐aging,” that is, the chronic activation of macrophages, since it was first described (Franceschi et al., 2000; Prattichizzo, Bonafe, Olivieri, & Franceschi, 2016). Macrophages are a widely distributed, heterogeneous, plastic cell population, with central roles as effectors and mediators of the innate and adaptive immune response (Mosser & Edwards, 2008; Wynn, Chawla, & Pollard, 2013; Zhou et al., 2014). Several studies in humans and animals have reported age‐related alterations in different macrophage functions, such as phagocytic activity, pro‐inflammatory cytokine secretion, and antigen presentation (Jackaman et al., 2017; Sebastian, Espia, Serra, Celada, & Lloberas, 2005). Furthermore, a loss of macrophages has been associated with an improved inflammation‐induced pathology and survival after systemic immunostimulation (Bouchlaka et al., 2013), and with reduced neurodegeneration (Yuan et al., 2018) in murine models of aging.

Endogenous glucocorticoids (GCs) are central regulators of immune functions. They can exert their action by binding to the widely expressed glucocorticoid receptor (GR) through genomic mechanisms (transactivation, transrepression, and composite glucocorticoid response element‐binding), or nongenomic effects (Cain & Cidlowski, 2017). GC secretion is regulated in a circadian manner and in response to stress by the hypothalamic–pituitary–adrenal (HPA) axis, whose function is dysregulated with advancing age (Gupta & Morley, 2014). However, the role of systemic and local GC production during aging processes is largely unknown.

Due to the importance of macrophage activation and GC responses in the aged immune system, we aimed to evaluate the age‐associated changes in GC metabolism in the myeloid compartment of mice in order to improve the current understanding of “macroph‐aging.”

## 2 RESULTS

### 2.1 Inflammation in aged mice is associated with decreased serum GC levels

**FIGURE 1:**
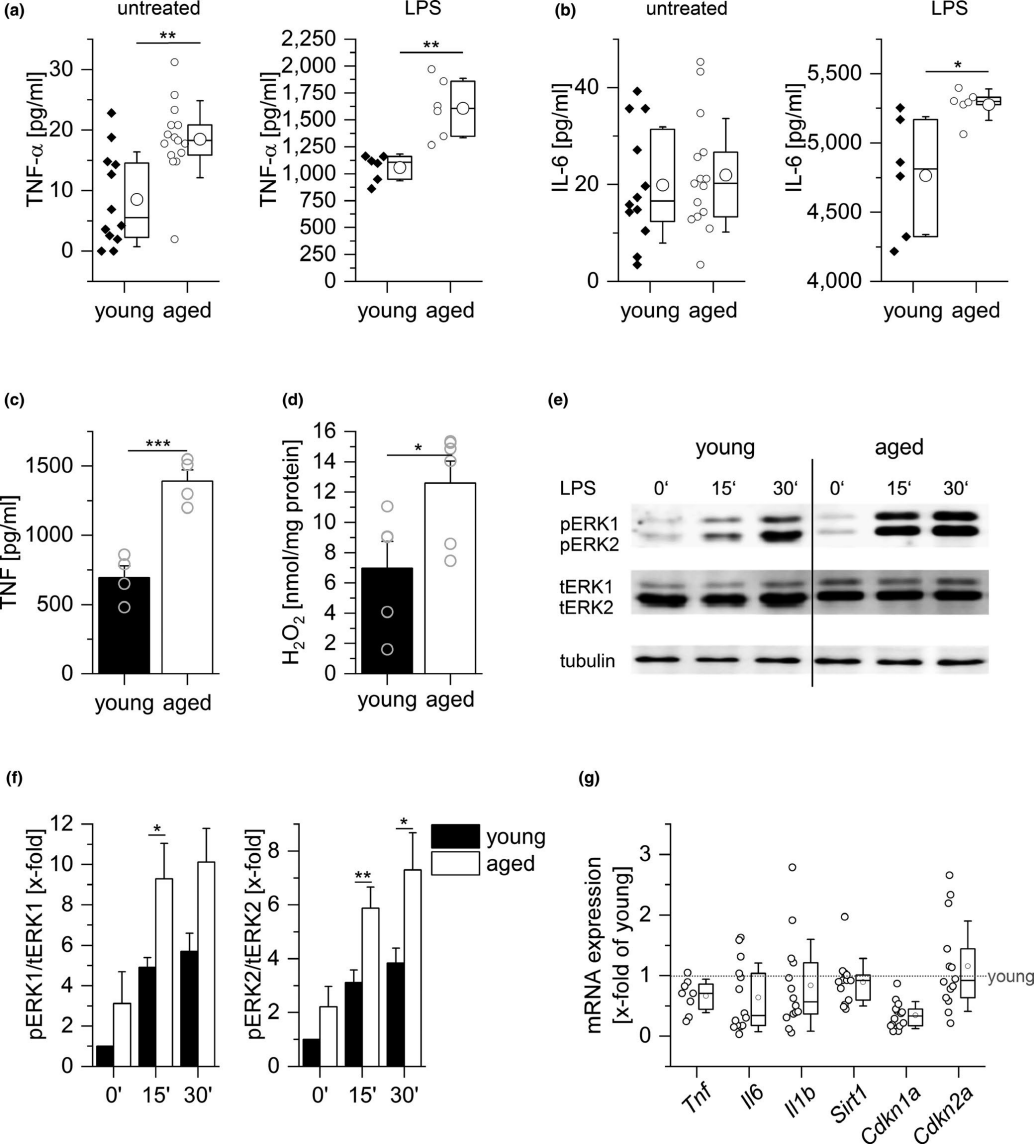
Inflammation in aged mice. Levels of TNF‐α (a) and IL‐6 (b) in sera from young (10 weeks) and aged (20–22 months) C57BL/6 mice as determined by ELISA; untreated: *n* = 12 (young) or *n* = 15 (aged), LPS (5 mg/kg BW, 4 hr): *n* = 6. (c) PMs obtained from young and aged mice were stimulated with LPS (100 ng/ml, 4 hr), and TNF was quantified in the supernatants by bioassay (*n* = 4, triplicates). (d) H_2_O_2_ production was measured in PM supernatants by homovanillic acid assay (young: *n* = 5, aged: *n* = 6). (e, f) ERK phosphorylation after LPS treatment (100 ng/ml) was examined by Western blot. Total ERK (tERK) and tubulin served as loading controls. (e) One representative blot out of six is shown. (f) pERK1/2 signal intensities were normalized to total ERK1/2. Values for untreated cells from young mice were set as 1 (*n* = 6 in replicates). (g) Gene expression in PMs from young and aged mice was determined by qPCR and normalized against the housekeeping gene (Ppia). Values for PMs from young mice were set as 1 (n = 14). Box plots show the 25–75th percentiles (box), mean (square), median (line), and *SD* (whiskers). Bars show the mean ± *SEM*, and circles within bars indicate the mean of each individual experiment. *p < .05, **p < .01, ***p < .001 by two‐tailed *t* test (a–c), Mann–Whitney *U* test (d, g), or ANOVA with Bonferroni's post hoc test (f)

Previous studies demonstrated that levels of circulating pro‐inflammatory mediators, such as tumor necrosis factor (TNF)‐α, interleukin (IL)‐6, and C‐reactive protein (CRP), are elevated in aged individuals, a circumstance which correlates with increased risk of morbidity and mortality (Bandaranayake & Shaw, 2016; Minciullo et al., 2016). In line with these findings, we observed a pro‐inflammatory phenotype in aged C57BL/6 mice when compared with their young counterparts. This phenotype was characterized by increased basal TNF‐α and LPS‐induced IL‐6 and TNF‐α serum levels (Figure 1a,b). In peritoneal macrophages (PMs) isolated from aged animals, a higher production of LPS‐induced TNF and H_2_O_2_ was found. In addition, extracellular‐signal‐regulated kinase (ERK) 1/2 activation, which is critically involved in cytokine production in macrophages (Hoppstädter et al., 2015, 2016), was increased (Figure 1c–f).

Senescent cells (SCs) are characterized by elevated cytokine production, upregulation of the cyclin‐dependent kinase inhibitors p16 (*Cdkn2a*) and p21 (*Cdkn1a*), and downregulation of the senescence suppressor sirtuin‐1 (*Sirt1*) (Lee, Lee, Lee, & Min, 2019; Tchkonia, Zhu, van Deursen, Campisi, & Kirkland, 2013; van Deursen, 2014). None of these effects were observed in PMs obtained from aged mice, indicating that these macrophages do not resemble SCs (Figure 1g).

**FIGURE 2:**
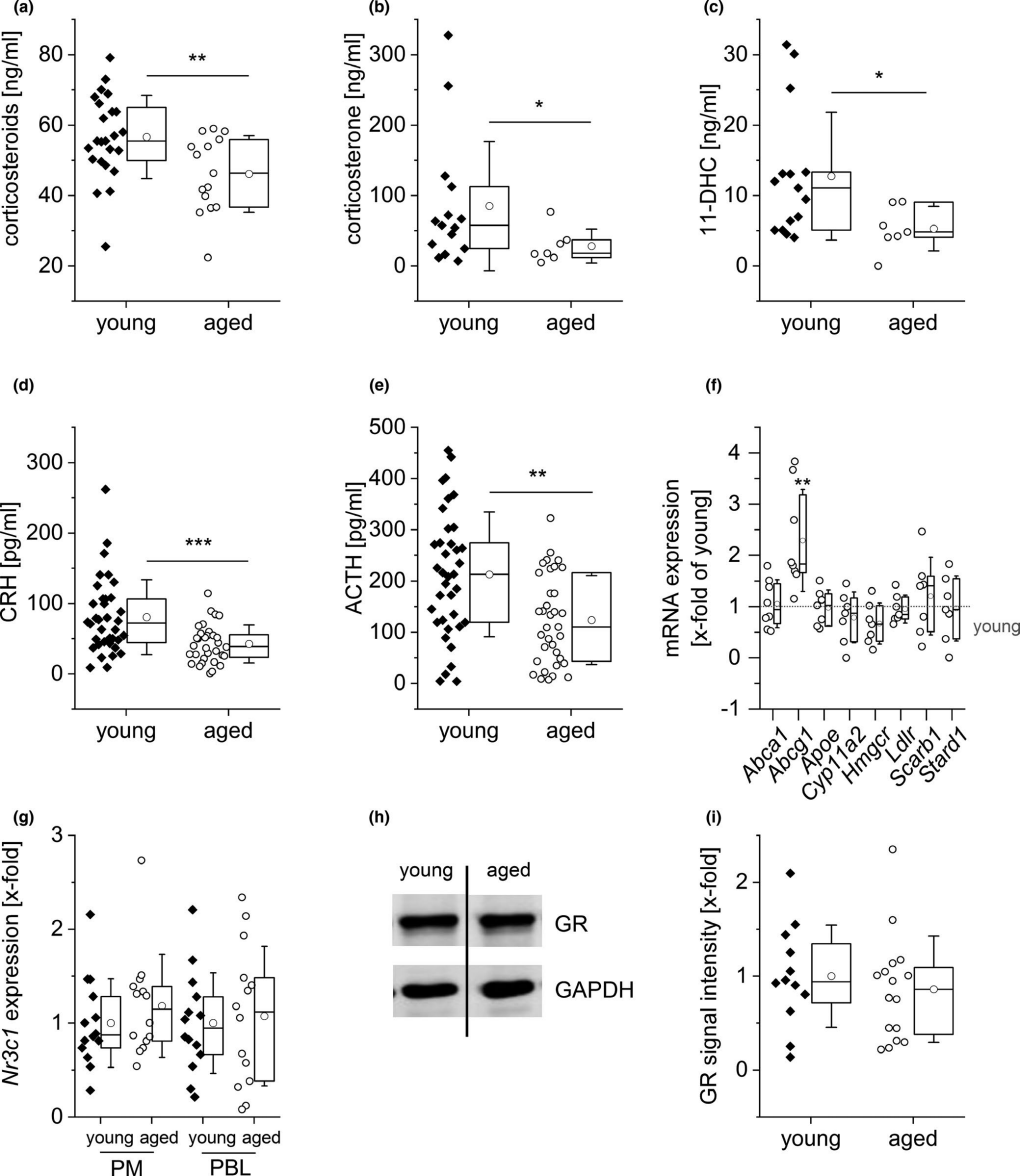
Decreased GC levels in aged mice. (a) Total serum corticosteroid levels were quantified in sera of young (*n* = 24) and aged (*n* = 15) mice by ELISA. (b, c) Corticosterone (b) and 11‐dehydrocorticosterone (11‐DHC, c) concentrations were determined in sera of young (*n* = 15) and aged (*n* = 7) mice by LC‐HRMS/MS. (d, e) CRH (d) and ACTH (e) levels were determined in sera of young (*n* = 34) and aged (*n* = 33) mice by ELISA. (f) Expression levels of genes involved in cholesterol homeostasis and corticosteroid biosynthesis were measured in adrenal glands from young (*n* = 12) and aged (*n* = 8) mice by qPCR, normalized against the housekeeping gene (*Csnk2a2*), and expressed as x‐fold of samples from young mice. (g) Expression of GR mRNA (*Nr3c1*) in PMs and PBLs from young and aged mice (*n* = 14). Data were normalized against the housekeeping gene (*Ppia*) and are expressed as fold change of young. (h, i) GR protein levels were measured by Western blot in PMs obtained from young (*n* = 12) and aged (*n* = 16) mice. (h) Representative blot. (i) GR signal intensities were normalized to the loading control GAPDH and are expressed as *x*‐fold of young. Box plots show the 25–75th percentiles (box), mean (circle), median (line), and *SD* (whiskers). *p < .05, **p < .01, ***p < .001 by two‐tailed *t* test (a, d, e) or Mann–Whitney *U* test (b, c, f)

Serum analyses by ELISA showed significantly reduced corticosteroid levels in aged mice (Figure 2a). Due to known cross‐reactivities, however, conventional antibody‐based detection methods for corticosteroids do not allow to distinguish between inactive cortisone/11‐dehydrocorticosterone (11‐DHC) and active cortisol/corticosterone. Thus, we developed an LC‐HRMS/MS‐based method to quantify the serum levels of the main GCs in rodents (Gong et al., 2015), that is, active corticosterone and inactive 11‐DHC, in sera from young and aged mice. Aged mice displayed decreased serum levels of both corticosteroids compared with young animals. While the mean of total GC and 11‐DHC levels was reduced by half, levels of active corticosterone decreased by two‐thirds (Figure 2b,c).

The synthesis of GCs within the adrenal cortex is regulated via the release of corticotropin‐releasing hormone (CRH) from the hypothalamus, which stimulates the secretion of adrenocorticotropic hormone (ACTH) from the anterior pituitary gland (Gupta & Morley, 2014). Serum levels of both CRH and ACTH were reduced in aged animals (Figure 2d,e). The expression of genes involved in the cholesterol homeostasis or corticosteroid synthesis within the adrenal gland remained unchanged, except that the cholesterol efflux mediator ATP‐binding cassette transporter G1 (*Abcg1*) was upregulated (Figure 2f). Taken together, these findings suggest an HPA axis dysfunction in aged mice.

### 2.2 GC homeostasis in aged cells and tissues

Irrespective of serum GC levels, the cellular GC responsiveness is ultimately determined by the intracellular availability of active GCs, as well as the expression of the GR (*Nr3c1*) (Cain & Cidlowski, 2017; Oakley & Cidlowski, 2013). However, GR levels remained unchanged with aging in PMs, peripheral blood leukocytes (PBLs), liver, lung, and lymphoid tissues (Figure 2g‐i  and Figure S1).

**FIGURE 3:**
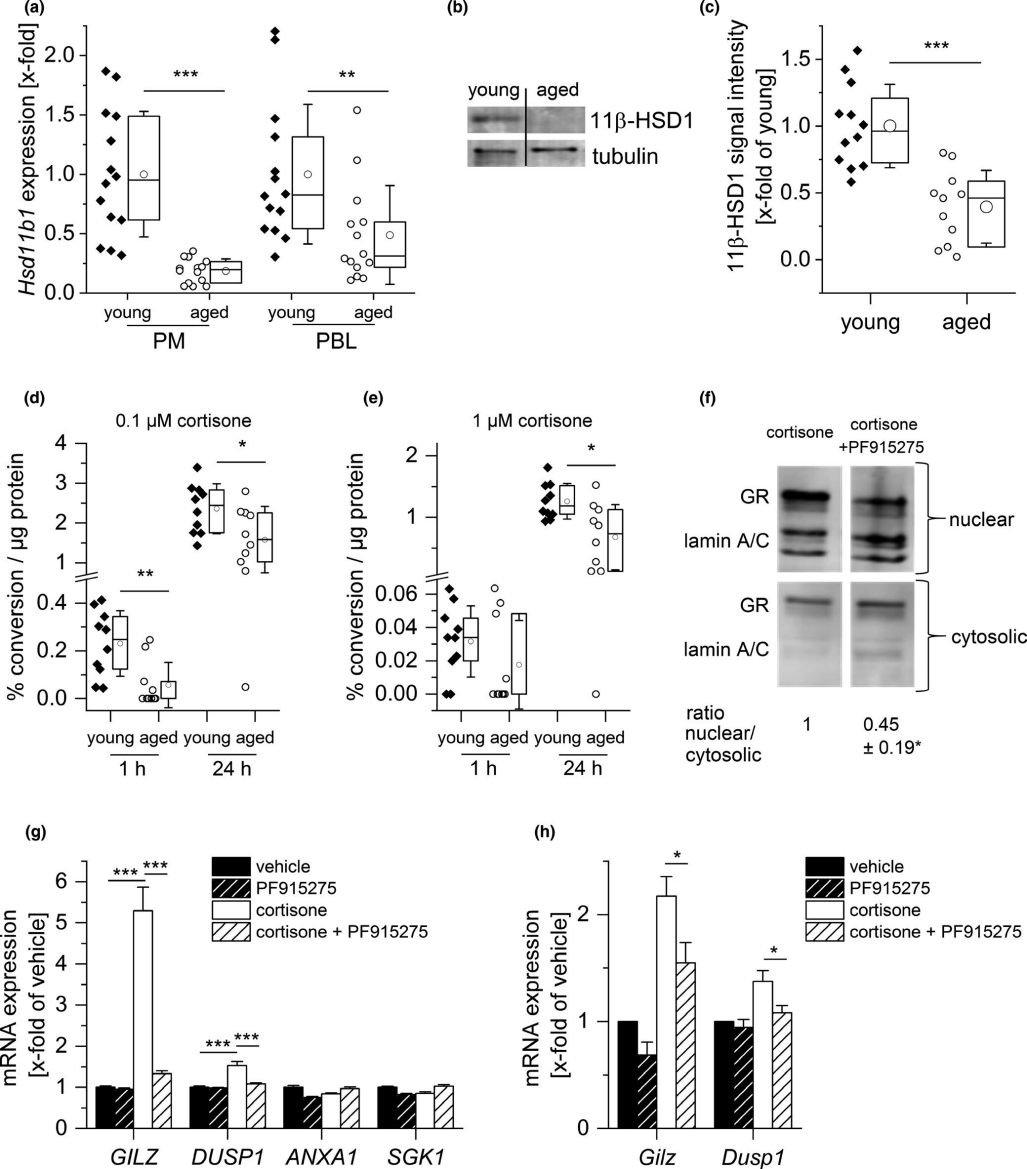
Alterations in GC metabolism in aged immune cells. (a) *Hsd11b1* expression in PMs and PBLs from young and aged mice (*n* = 14) was measured by RT‐qPCR, normalized to *Ppia*, and expressed as x‐fold of young. (b, c) 11β‐HSD1 protein expression was measured in PMs (young: *n* = 12, aged: *n* = 11). (b) Representative blot. (c) Densitometric analysis, normalized to the housekeeping protein tubulin. Values for young mice were set as 1. (d, e) PMs isolated from young and aged animals (*n* = 10) were incubated for 1 or 24 hr with 0.1 µM (d) or 1 µM (e) cortisone‐D8, and the conversion to cortisol‐D8 was measured in supernatants by LC‐HRMS/MS. Conversion percentages were normalized to total protein content. (f, g) THP‐1 macrophages were treated with either vehicle (0.1% DMSO) or the 11β‐HSD1 inhibitor PF915275 (100 nM) for 1 hr, followed by cortisone treatment (100 nM) for 4 hr. (f) GR content in nuclear and cytosolic fractions was analyzed by Western blot. Lamin (A/C) was used as an indicator for the presence or absence of nuclear proteins. One representative blot is shown. Densitometric quantification data are presented as x‐fold of the nuclear/cytosolic GR ratio in cortisone‐treated cells (*n* = 4, *p < .05 versus cortisone‐treated cells). (g) GR‐dependent gene expression was analyzed by qPCR and normalized to the housekeeping gene *ACTB*. Data are presented as x‐fold of vehicle‐treated cells (*n* = 3, triplicates). (h) PMs obtained from young mice were treated with either vehicle (0.1% DMSO) or PF915275 (100 nM) for 1 hr, followed by cortisone treatment (100 nM) for 4 hr. *Gilz* and *Dusp1* expression were analyzed by qPCR, normalized to the housekeeping gene *Ppia*, and expressed as x‐fold of vehicle control (*n* = 8). Box plots show the 25–75th percentiles (box), mean (circle), median (line), and *SD* (whiskers). Bars show the mean ± *SEM*. *p < .05, **p < .01, ***p < .001 by two‐tailed *t* test (a, c), one‐sample *t* test (f), Mann–Whitney *U* test (d, e), or ANOVA with Bonferroni's post hoc test (g, h)

It is well established that macrophages and other cells and tissues can generate active cortisol from inactive cortisone to locally amplify steroid action (Gilmour et al., 2006). We, therefore, determined gene expression levels of the enzyme 11β‐hydroxysteroid dehydrogenase type 1 (11β‐HSD1, *Hsd11b1*), which locally converts GCs to their active form. We found a significantly lower *Hsd11b1* gene expression in PM and PBLs from aged mice (Figure 3a), and also in the liver, where the enzyme is most abundant (Figure S2A). Accordingly, 11β‐HSD1 protein levels were reduced in aged PMs (Figure 3b,c). On the other hand, the gene expression of *Hsd11b2*, the isozyme that exerts dehydrogenase (cortisol/corticosterone to cortisone/11‐DHC) activity, remained unchanged in PMs and PBLs from aged mice compared with young controls (Figure S2b).

Transcription of *Hsd11b1* is regulated by members of the CCAAT/enhancer‐binding protein (C/EBP) family of transcription factors (Chapman, Holmes, & Seckl, 2013). To determine whether these upstream regulators of 11β‐HSD1 were altered in aged PM and PBL, mRNA levels of C/EBPα and C/EBPβ were measured. Both genes were significantly downregulated in PMs from aged animals. In PBLs, a similar tendency for *Cebpa* expression was observed, while the expression of *Cebpb* remained unchanged (Figure S2c,d).

Since 11β‐HSD1 regulates the intracellular availability of active GCs, age‐related changes in 11β‐HSD1 reductase activity were evaluated by measuring the conversion rate of deuterated cortisone to cortisol in isolated young and aged PMs by LC‐HRMS/MS. The downregulation of the enzyme was indeed paralleled by lower levels of intracellular conversion of cortisone to cortisol (Figure 3d,e). These findings suggested that reduced 11β‐HSD1 expression translates into less corticosterone available to activate the GR in aging macrophages in the in vivo setting.

To assess the functional consequences of the loss of 11β‐HSD1, THP‐1 macrophages were pretreated with the 11β‐HSD1 inhibitor PF915275, followed by an analysis of GR translocation upon cortisone treatment. 11β‐HSD1 inhibition abrogated GR translocation (Figure 3f) and reduced the cortisone‐induced expression of GR‐responsive genes, particularly the anti‐inflammatory mediator glucocorticoid‐induced leucine zipper (GILZ, *Tsc22d3*) (Figure 3g). Similar results were obtained when using PMs from young mice instead of THP‐1 cells (Figure 3h).

### 2.3 GILZ downregulation as a feature of inflammaging

**FIGURE 4:**
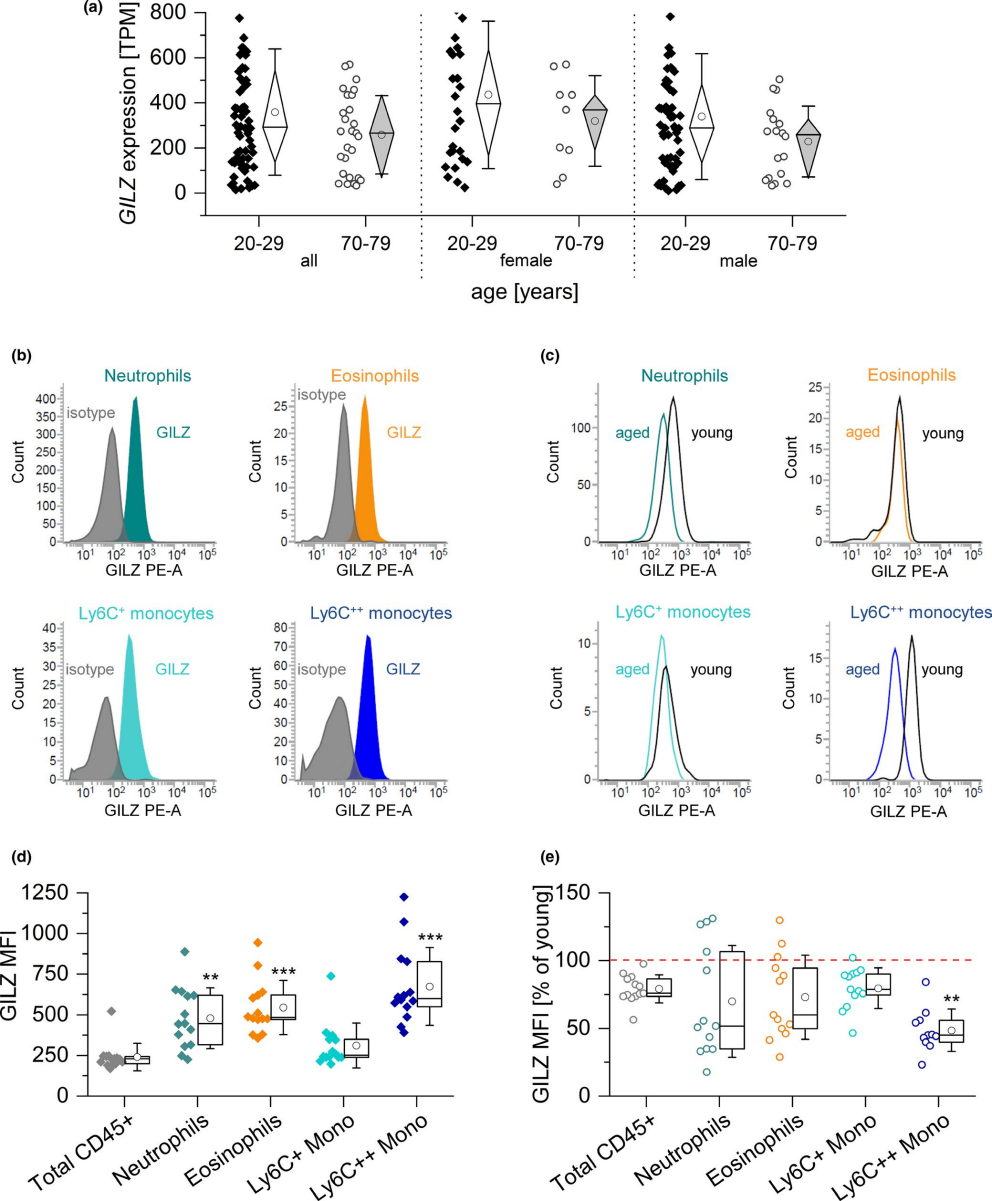
GILZ expression in human and murine blood samples from young and aged individuals. (a) GILZ expression in human blood samples. Expression data were retrieved from the GTEx database and are shown as TPM (transcripts per million) (20–29 years: 27 ♀, 57 ♂; 70–79 years: 9 ♀, 18 ♂). (b) Representative histograms showing GILZ expression in myeloid subsets in young mice. Colored: GILZ signal; gray: isotype control. (c) Representative histograms showing GILZ signals in cells from aged and young mice. Gray line: young; colored line: aged. (d) Background‐subtracted GILZ median fluorescence intensity (MFI) in myeloid blood cells from young mice (*n* = 14). **p < .01, ***p < .001 compared with MFI values for total CD45^+^ cells by ANOVA with Bonferroni's post hoc test. (e) GILZ expression in myeloid subsets of aged mice (*n* = 13). The GILZ MFI of young cells was set as 100% for each subset.Box plots show the 25‐75th percentile (box), mean (circle), median (line), and SD (whiskers). *p < .05, **p < .01 compared with the same subset in young animals by Mann–Whitney *U* test. Mono: monocytes

Our data suggested GILZ expression as an indicator of altered GC homeostasis. Thus, we analyzed its expression in human blood samples from the publicly available GTEx database (version 8). GILZ tended to be downregulated in human blood samples from aged individuals, although the expression levels showed high inter‐individual variability (Figure 4a).

To further elucidate whether GILZ expression was affected by the aging process, we measured its expression in various murine cells and tissues. Based on a multi‐color flow cytometric analysis (Figure 4b,c and Figure S3), we found that GILZ was highly expressed in myeloid peripheral blood cells from young animals (Figure 4d), and was significantly downregulated in monocytes from aged mice (Figure 4e).

**FIGURE 5:**
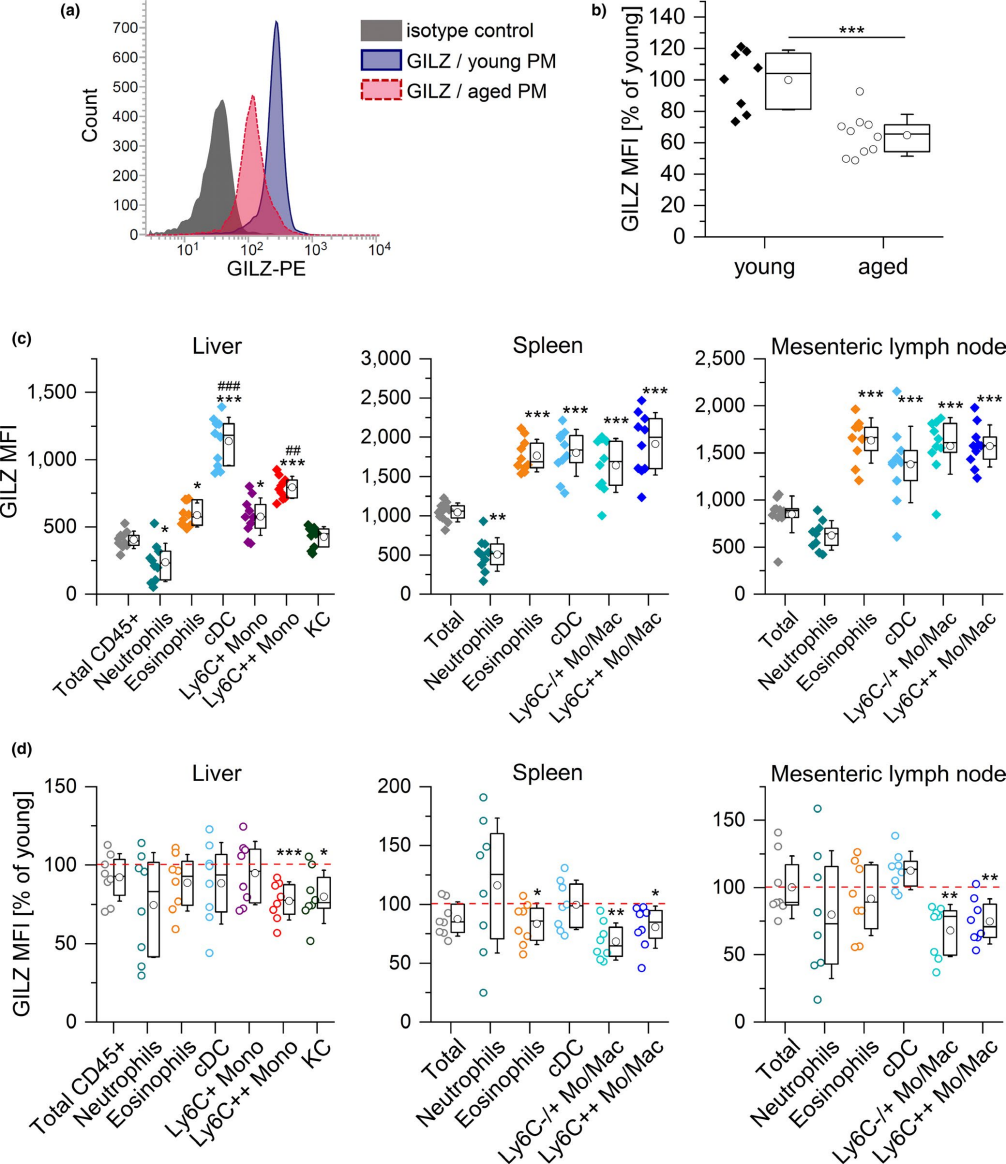
GILZ expression in myeloid cells from young and aged mice. (a, b) GILZ expression in PMs from young and aged mice was determined by flow cytometry. (a) Representative histogram. (b) Background‐subtracted GILZ median fluorescence intensity (MFI) (young: *n* = 8, aged: *n* = 10). (c) GILZ MFI in myeloid cells in the liver and lymphoid tissues of young mice (*n* = 10). ***p < .001 compared with MFI values for total CD45^+^ (liver) or total cells (spleen and lymph node), ^##^ *p* < .01, ^###^ *p* < .001 compared with all other subsets (ANOVA with Bonferroni's post hoc test. (d) GILZ expression in myeloid subsets of aged mice. The GILZ MFI of young cells was set as 100% for each subset (*n* = 8). *p < .05, **p < .01, ***p < .001 compared with the same subset in young animals by Mann–Whitney *U* test. Box plots show the 25–75th percentiles (box), mean (circle), median (line), and *SD* (whiskers). Mono: monocytes; Mo/Mac: monocytes and macrophages; cDC: classical dendritic cells; KC: Kupffer cells

Furthermore, flow cytometric quantification of GILZ expression in PMs obtained from aged mice showed reduced GILZ levels when compared with cells derived from young animals (Figure 5a,b). GILZ was highly expressed in myeloid cells in livers, spleens, and mesenteric lymph nodes from young mice (Figure 5c, Figures S4 and S5). In tissues from aged animals, we observed a downregulation of GILZ, which was particularly evident in the monocyte and macrophage compartment (Figure 5d, Figures S4 and S5).

**FIGURE 6:**
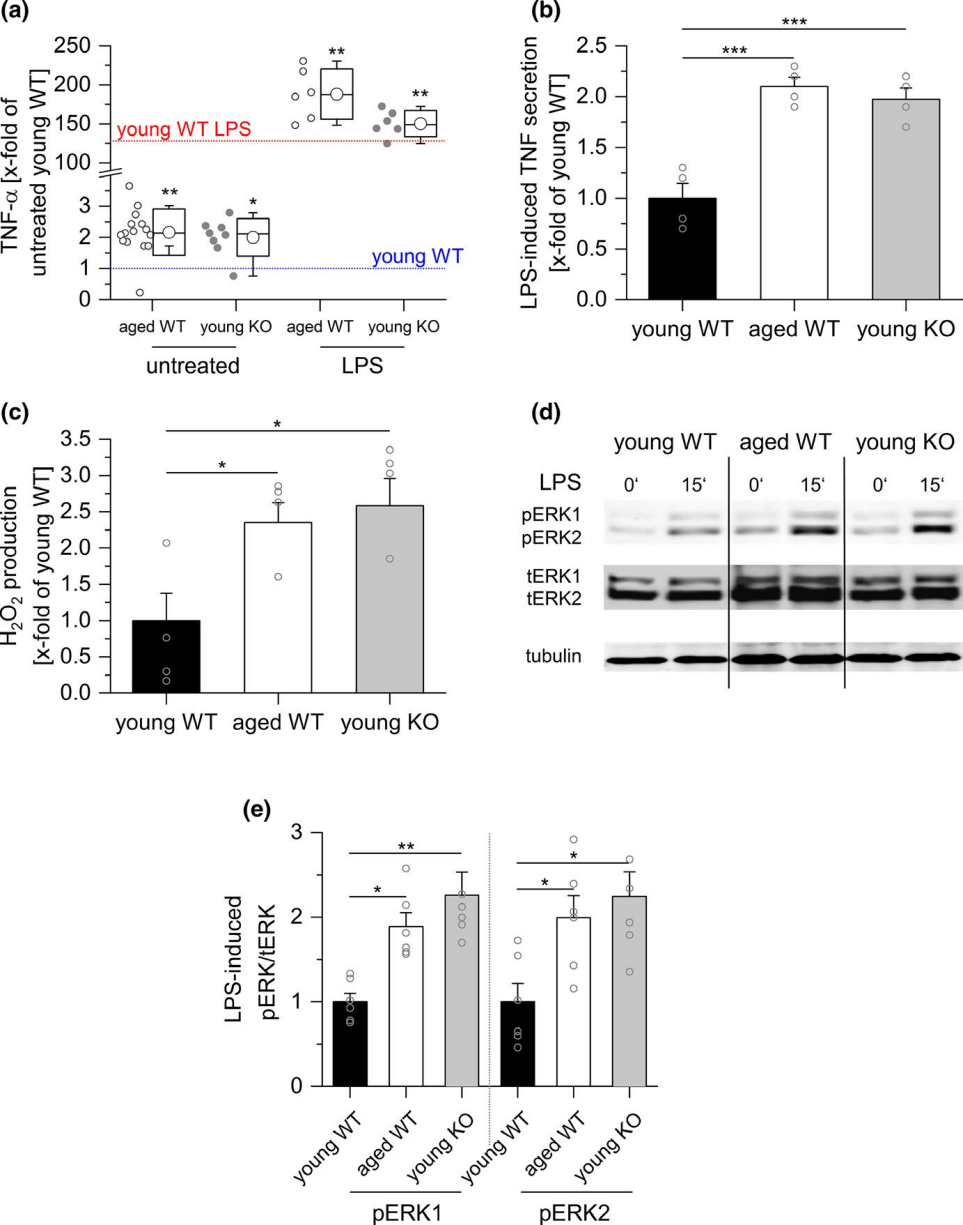
GILZ knockout mimics inflammaging. (a) Serum TNF‐α levels in young GILZ knockout (KO) mice were determined by ELISA and compared with serum concentrations in young and aged wild‐type (WT) mice (see Figure 1). The mean of the values for young untreated WT mice was set as 1 (blue line). The red line indicates the mean TNF‐α level in LPS‐treated young WT mice, expressed as x‐fold of untreated young WT mice. Untreated young KO: *n* = 12, LPS‐treated young KO (5 mg/kg BW, 4 hr): *n* = 6. *p < .05, **p < .01 compared with equally treated young WT animals by ANOVA with Bonferroni's post hoc test. (b) PMs obtained from young and aged WT, as well as young KO mice, were stimulated with LPS (100 ng/ml, 4 hr), and TNF production was assessed by bioassay. Values for young WT mice were set as 1 (*n* = 4, triplicates). (c) H_2_O_2_ production was measured in PM supernatants by homovanillic acid assay (young WT and KO: *n* = 5, aged: *n* = 6). (d, e) ERK phosphorylation after LPS treatment (100 ng/ml, 15') was analyzed by Western blot. (d) One representative blot out of six is shown. Total ERK (tERK) and tubulin served as loading controls. (e) Densitometric analysis of LPS‐induced ERK phosphorylation. pERK1/2 signal intensities were normalized to tERK1/2 and expressed as x‐fold of young WT. Box plots show the 25–75th percentiles (box), mean (circle), median (line), and *SD* (whiskers). Bars show the mean ± *SEM*, and circles within bars indicate the mean of each experiment. *p < .05, **p < .01, ***p < .001 by ANOVA with Bonferroni's post hoc test

GILZ represents a potent endogenous suppressor of inflammatory responses (Bereshchenko, Migliorati, Bruscoli, & Riccardi, 2019; Hoppstädter & Kiemer, 2015; Ronchetti, Migliorati, Bruscoli, & Riccardi, 2018). Thus, we hypothesized that the loss of GILZ in aging macrophages might contribute to inflammaging. To test this hypothesis, we compared young mice bearing a myeloid‐specific GILZ knockout (KO) with young and aged wild‐type (WT) mice. The phenotype of aged mice was mimicked in young GILZ KO mice: Both baseline and LPS‐induced serum TNF‐α levels were elevated in both groups (Figure 6a). Furthermore, we analyzed aspects of cell activation in PMs from young WT, old WT, and young KO mice (Figure 6b–e). TNF production, generation of reactive oxygen species, and ERK phosphorylation were increased both in macrophages from aged WT as well as young KO animals, indicating that the downregulation of GILZ might be a central feature of inflammaging.

## 3 DISCUSSION

Aging, defined as the functional decline that occurs during the lifespan of an organism, constitutes a risk factor for several human pathologies (Lopez‐Otin, Blasco, Partridge, Serrano, & Kroemer, 2013). For instance, the prevalence and mortality associated with CVD are expected to grow exponentially, as the world population continues to age (Costantino, Paneni, & Cosentino, 2016). Inflammation is known to promote aging processes (Franceschi et al., 2018; Kennedy et al., 2014). Indeed, longitudinal studies have shown a correlation between inflammation and longevity, capability, and cognition in the aged population (Arai et al., 2015). The changes in the immune system associated with aging, globally known as immunosenescence, affect both innate and adaptive immunity. Rather than solely detrimental, such changes represent an adaptive/remodeling response that results in dysregulated homeostasis not only of immunity, but also of other systems that influence and are influenced by the immune system, such as the nervous and endocrine systems (Fulop et al., 2017). The chronic, low‐grade systemic inflammatory state observed with advancing age, termed inflammaging (Franceschi et al., 2000), involves functional alterations of immune cells as a consequence of different mechanisms, including cellular senescence, oxidative stress, mitochondrial dysfunction, defective autophagy and mitophagy, inflammasome activation, and dysbiosis (Franceschi et al., 2018). Population studies have demonstrated that aged individuals show elevated circulating levels of pro‐inflammatory mediators/markers, such as TNF‐α, IL‐6, and CRP, which correlate with increased risk of morbidity and mortality (Bandaranayake & Shaw, 2016; Minciullo et al., 2016).

While the mechanisms responsible for the initiation of inflammaging are still widely unknown, an increasing body of evidence suggests that the accumulation of SCs is critically involved in this process. Factors secreted by cells with a senescence‐associated secretory phenotype (SASP), that is, pro‐inflammatory cytokines and chemokines, matrix metalloproteinases, and growth factors, have been shown to contribute to age‐related diseases. This pro‐inflammatory program is thought to be activated in order to initiate the immune‐mediated clearance of SCs (Tchkonia et al., 2013; van Deursen, 2014). Noticeably, macrophage depletion by clodronate treatment has been reported to induce a drastic reduction of age‐associated inflammatory responses to systemic immunostimulation in aged mice (Bouchlaka et al., 2013). These findings strongly support the hypothesis that macrophages play a vital role in aging processes, a phenomenon previously termed “macroph‐aging” (Franceschi et al., 2000). In line with these assumptions, our data show an inflammatory phenotype in old versus young adult mice, as indicated by elevated TNF‐α and IL‐6 serum levels, increased TNF‐α and reactive oxygen species production in macrophages from aged animals, and increased ERK phosphorylation upon LPS stimulation.

The global pro‐inflammatory state, however, is not the only determinant of successful/unsuccessful aging, as a compensatory, anti‐inflammatory response, is observed in parallel to inflammaging, particularly in very long‐lived individuals (centenarians). The mediators associated with anti‐inflammaging include transforming growth factor (TGF)‐β, IL‐10, dehydroepiandrosterone (DHEA), and cortisol (Baylis, Bartlett, Patel, Roberts, 2013; Minciullo et al., 2016). It is considered that, ultimately, the ability of the anti‐inflammatory network to cope with and modulate chronic inflammation is determinant either to attain healthy aging and longevity or for the onset of chronic inflammatory diseases.

The synthesis of GCs within the adrenal cortex is regulated by the HPA axis via the release of CRH and arginine vasopressin (AVP) from the hypothalamus. Subsequently, CRH and AVP stimulate the secretion of ACTH from the anterior pituitary gland (Gupta & Morley, 2014). Serum levels of both CRH and ACTH were reduced in aged animals. The expression of genes involved in the regulation of intracellular cholesterol levels or corticosteroid synthesis within the adrenal gland remained unchanged, except that the ATP‐binding cassette transporter G1 (*Abcg1*) was upregulated. Although *Abcg1* is mainly described to mediate cholesterol efflux, there might be additional functions within the adrenal cortex, as suggested by a mild glucocorticoid insufficiency observed in *Abcg1* knockout mice (Hoekstra et al., 2019). In summary, these observations suggest a dysfunction of the HPA axis with advanced age.

The involvement of GC signaling and metabolism in chronic inflammation and stress‐related disorders, two phenomena that occur with aging, has been a topic of interest for the last decades (Herriot, Wrosch, Gouin, & Miller, 2017). Literature reports indicate associations between both higher and lower GC levels and negative health outcomes (Gaffey, Bergeman, Clark, & Wirth, 2016; Raison & Miller, 2003). In rodents, an increase (Kizaki et al., 2002), no differences (Morano, Vazquez, & Akil, 1994) and, more recently, a decrease (Zambrano, Reyes‐Castro, & Nathanielsz, 2015) in serum corticosteroids with age have been reported. However, investigations on total corticosteroid concentrations in blood or urine do not consider that aging might affect the glucocorticoid metabolism.

Cellular GC responsiveness is determined by the intracellular availability of active GCs, as well as the expression of GRs. Although a previous study reported elevated GR mRNA expression in peritoneal exudate cells from aged mice (Kizaki et al., 1998), our findings suggest that alterations in GC homeostasis from PMs are not associated with altered GR expression but with reduced intracellular activation of 11‐DHC by 11β‐HSD1.

Transcription of *Hsd11b1* is controlled by members of the C/EBP family of transcription factors. Interestingly, C/EBPα and C/EBPβ themselves are GC‐inducible (Ayala‐Sumuano et al., 2013; Chapman et al., 2013), suggesting a feed‐forward loop to further amplify local glucocorticoid signaling. In our setting, both *Cebpa* and *Cebpb* were downregulated in aged macrophages, implying that this regulatory circuit might be disrupted in aged mice.

There might be different functional implications of blunted 11β‐HSD1 signaling in aged macrophages: It has been shown that 11β‐HSD1‐deficient mice are more susceptible to endotoxemia, and macrophages derived from these mice are hyperresponsive to LPS stimulation (Zhang & Daynes, 2007). Loss of 11β‐HSD1 in macrophages has also been associated with delayed phagocytic capacity (Gilmour et al., 2006).

In our hands, pharmacological inhibition of 11β‐HSD1 resulted in a decreased expression of GC‐responsive genes, particularly *GILZ*. GILZ was first identified as a dexamethasone‐inducible gene in murine thymocytes (D'Adamio et al., 1997) and has been suggested to play a critical role in the anti‐inflammatory activity of GCs (Bereshchenko et al., 2019; Ronchetti et al., 2018). On the molecular level, GILZ facilitates its anti‐inflammatory activity by binding to the pro‐inflammatory transcription factors NF‐κB and activator protein (AP)‐1, thereby preventing their nuclear translocation (Ayroldi et al., 2001; Bruscoli et al., 2018). GILZ also interferes with mitogen‐activated protein kinase (MAPK) signaling, for example, by binding to Ras/Raf, resulting in the inhibition of downstream MAP kinases, such as ERK (Hoppstädter et al., 2015; Ricci et al., 2019). In this manner, GILZ plays a vital role in macrophage activation (Hoppstädter et al., 2012, 2015; Hoppstädter, Diesel, et al., 2019; Hoppstädter & Kiemer, 2015). Under inflammatory conditions, repression of GILZ expression represents a regulatory mechanism that prolongs and/or increases pro‐inflammatory responses in human and murine macrophages (Hoppstädter et al., 2012, 2015). In contrast, the immunosuppressive phenotype of macrophages differentiated in the presence of M‐CSF and IL‐10 is associated with elevated *GILZ* expression (Seif, Hoppstädter, Breinig, & Kiemer, 2017). Based on these observations and the findings on GC homeostasis in aged mice shown within the present study, we hypothesized that GILZ might be downregulated during inflammaging. We indeed observed reduced GILZ levels in blood monocytes, PMs, as well as monocytes and macrophages in various tissues, from aged mice. The analysis of publicly available GTEx datasets showed that GILZ also tended to be downregulated in human blood samples from aged individuals, although the expression levels showed high inter‐individual variability. This may be explained by the fact that GILZ expression follows a circadian rhythm (Ayyar, Almon, Jusko, & DuBois, 2015) and can also be influenced by medication, most notably GCs and statins (Bereshchenko et al., 2019; Hoppstädter et al., 2020).

To assess the functional implications of GILZ downregulation, we compared young WT, old WT, and young myeloid‐specific GILZ KO mice, and found that GILZ KO mimicked the inflammaging phenotype. These data support the hypothesis that the loss of GILZ in aging macrophages fuels the process of tissue inflammaging. Of note, other effectors of GC responses, such as dual‐specificity phosphatase 1 (*Dusp1*) (Hoppstädter & Ammit, 2019), may also contribute to the overall effect.

In summary, the present study suggests a link between GC metabolism and the age‐related inflammatory status that was unknown to date. The reduced circulating corticosterone levels, together with a decreased availability of active GCs within cells as a consequence of diminished 11β‐HSD1 expression, might contribute to the imbalance between pro‐ and anti‐inflammatory signaling in aged macrophages, thus promoting inflammaging.

## 4 EXPERIMENTAL PROCEDURES

### 4.1 Materials

Fetal bovine serum (FBS, #F7524), RPMI1640 (#R0883), trypsin/EDTA (#T3924), Accutase (#L11‐007‐1), penicillin/streptomycin (#P433), and glutamine (#G7513) were from Sigma‐Aldrich. Anti‐p44/42 (ERK1/2) mouse antibody (L34F12, #4696S) and anti‐phospho‐p44/42 MAPK (Thr202/Tyr204) rabbit mAbs (20G11, #4376S) were obtained from Cell Signaling Technology. The anti‐tubulin antibody (#T9026) was obtained from Sigma‐Aldrich. The anti‐GAPDH antibody (OTI2D9, #TA802519) was from OriGene. Anti‐rabbit IRDye 680‐ and anti‐mouse/anti‐goat IRDye 800‐conjugated secondary antibodies were from LI‐COR Biosciences (#926‐68071, #926‐32210, #926‐32214). The anti‐rabbit IRDye 800‐conjugated secondary antibody was obtained from Rockland (#612‐132‐120). Antibodies for flow cytometry, that is, anti‐CD16/CD32 (Fcγ III/II Receptor) (2.4G2, #553142), anti‐CD45‐FITC (104, #561874), anti‐CD45R/B220‐FITC (RA3‐6B2, #553087), anti‐NK‐1.1‐Brilliant Violet™ 510 (PK136, #563096), anti‐CD11c‐APC (HL3, #550261), anti‐CD11b‐APC‐R700 (M1/70, #564985), anti‐CD14 Brilliant Violet™ 510 (rmC5‐3, #740125), anti‐I‐A/I‐E‐PerCP‐Cy™ 5.5 (M5/114.15.2, #562363), anti‐F4/80‐Brilliant Violet™ 421 (T45‐2342, #565411), anti‐Ly6G‐APC‐H7 (1A8, #565369), anti‐Ly6C‐Brilliant Violet™ 421 (AL‐21, #562727), and Brilliant stain buffer (#563794) were obtained from BD Biosciences. Anti‐GILZ‐PE (CFMKG15, #12‐4321‐82) and PE‐labeled rat IgG2aκ isotype control (eBR2a, #12‐4033‐82) were from eBioscience. Anti‐CD68‐Alexa594 (FA‐11, #137020) and the Zombie Yellow viability stain (#423104) were from BioLegend. The anti‐11β‐HSD1 polyclonal goat antibody (#AF3397‐SP) was from Abcam. Anti‐GR rabbit mAb (D8H2, #3660) and anti‐Lamin A/C rabbit polyclonal Ab (#2032) were from Cell Signaling.

The TNF‐α and IL‐6 ELISA kits were purchased from Cayman Chemical (#500850, #583371), the corticosterone ELISA kit (#ADI‐900‐097) was from Enzo, and the CRH (#CEA835M) and ACTH (#CEA836Mu) ELISA kits were from Cloud Clone. Ultrapure LPS from *Escherichia coli* K12 (#tlrl‐peklps) was obtained from InvivoGen. Phorbol 12‐myristate 13‐acetate (PMA, #524400) was from Cayman Chemical. MTT (# M5655), actinomycin D (#A9415), homovanillic acid (HVA, #H1252), and horseradish peroxidase (#P8250) were obtained from Sigma‐Aldrich. Murine M‐CSF (#130‐101‐704) and TNF‐α (#130–101–689) were obtained from Miltenyi Biotec. The 11β‐HSD1 inhibitor PF 915275 was obtained from Santa Cruz (#sc‐204182). DNA oligos were provided by Eurofins Genomics. The 5x HOT FIREPol^®^ EvaGreen^®^ qPCR Mix Plus was from Solis BioDyne (#08‐25). Other chemicals were obtained from either Sigma‐Aldrich or Carl Roth unless stated otherwise.

### 4.2 Mice

Mice were housed in a 12/12‐hr light/dark cycle with food and water ad libitum. Myeloid‐specific GILZ knockout (KO) mice were generated as previously described (Bruscoli et al., 2012; Hoppstädter et al., 2015). For aging studies, young (10 weeks) and aged (20–22 months) C57BL/6J mice were either not treated or treated with one intraperitoneal injection of 5 mg/kg LPS (#tlrl‐smlps; InvivoGen) or vehicle (endotoxin‐free DPBS, #TMS‐012‐A; Sigma‐Aldrich) for 4 hr before sacrifice (approval number GB 3‐2.4.2.2‐06/2016). Female mice were used for LPS injections. Groups of untreated mice comprised both male and female mice. No sex‐dependent differences in any of the readout parameters were observed (data not shown).

### 4.3 Cell culture

#### 4.3.1 Murine peritoneal macrophages

Peritoneal macrophages were isolated from young and aged C57BL/6J mice by washing the peritoneal cavity with cold PBS‐EDTA (137 mM NaCl, 2.7 mM KCl, 10.1 mM Na_2_HPO_4_, 1.8 mM KH_2_PO_4_, 5 mM Na_2_EDTA, pH 7.4). The fluid was collected and centrifuged for 10 min at 350 *g* and 4°C. Cells were resuspended in RPMI‐1640 medium supplemented with 10% FCS (FBS Good Forte, #P40‐47500; PAN‐Biotech), 100 U/ml penicillin, 100 µg/ml streptomycin, and 2 mM glutamine, seeded into a 35 mm cell culture dish, and allowed to adhere for 2 hr. Nonadherent cells were removed by washing with PBS, and PMs were detached with Accutase. The cell suspension was centrifuged, and the pellets were frozen at −80°C. Alternatively, cells were resuspended and plated in RPMI‐1640 medium without FCS and, after removing the nonadherent cells after 1 hr, treated as indicated. PMs were >95% pure as determined by flow cytometric analysis of F4/80 and CD68 expression (data not shown).

#### 4.3.2 Murine peripheral blood leukocytes

Whole blood from young and aged C57BL/6J mice was collected in PBS‐EDTA (5 mM EDTA in PBS) containing tubes and centrifuged at 500 *g* and 4°C for 20 min. The cell pellet was resuspended in 1 ml erythrocyte lysis buffer (155 mM NH_4_Cl, 10 mM KHCO_3_, 1 mM Na_2_EDTA) and incubated on ice for 15 min. After 5 min of centrifugation at 500 *g* and 4°C, the supernatant was discarded and the PBL pellet was frozen at −80°C until further use.

#### 4.3.3 Cell lines

THP‐1 (#TIB202) and L929 cells (#CRL‐6364) were obtained from ATCC and grown in standard medium (RPMI 1640, 10% FCS, 100 U/ml penicillin G, 100 μg/ml streptomycin, 2 mM glutamine). THP‐1 cells were differentiated into macrophage‐like cells by treatment with PMA (100 ng/ml) for 48 hr and kept in serum‐free medium for cortisone treatment.

### 4.4 Enzyme‐linked immunosorbent assay

Serum TNF‐α, IL‐6, corticosteroid, CRH, and ACTH levels were quantified in murine serum samples by ELISA as recommended by the supplier.

### 4.5 TNF bioassay

Murine TNF (TNF‐α/β) levels in cell culture supernatants were quantified by bioassay as previously described (Hoppstädter, Diesel, et al., 2019; Hoppstädter et al., 2015).

### 4.6 Homovanillic acid assay

H_2_O_2_ levels were measured using the homovanillic acid (HVA) assay, as described previously (Kessler et al., 2017). PMs were seeded at a density of approximately 2.5 × 10^5^ cells per well into a 24‐well plate. After 1 hr, cells were washed with PBS twice and 300 µl freshly prepared HVA solution (100 μM HVA, 4 U/ml horseradish peroxidase, dissolved in PBS containing Ca^2+^ and Mg^2+^; Sigma‐Aldrich) was added. Cells were then incubated at 37°C for 2 hr. 40 µl HVA stop buffer (0.1 M glycine, 0.1 M NaOH, 25 mM EDTA in water; Sigma‐Aldrich) were added to each well of a black 96‐well plate, and 260 μl of the extracellular HVA supernatant were added. The fluorescence (312 nm excitation, 420 nm emission) was determined using a SpectraMax M5e (Molecular Devices). A standard curve of H_2_O_2_ (0–5 µM) was run alongside the samples. Total cellular protein concentrations used for data normalization were determined by Pierce BCA protein assay (Thermo Fisher Scientific, #23225) according to the manufacturer's instructions.

### 4.7 Quantitative RT–PCR

Quantitative RT–PCR (qPCR) was performed as described previously (Dembek et al., 2017; Hoppstädter, Dembek, et al., 2019; Hoppstädter, Diesel, et al., 2019; Hoppstädter et al., 2015, 2016; Kessler et al., 2017). Total RNA from cells was isolated using the High Pure RNA Isolation Kit (#11828665001; Roche), following the manufacturer's instructions. Total RNA from murine tissue was isolated using the QIAzol lysis reagent (#79306; Qiagen) as recommended by the supplier. Residual genomic (g) DNA contamination was removed using the DNA‐free™ DNA Removal Kit (#AM1906; Thermo Fisher Scientific). To verify the absence of gDNA, a SINE‐PCR was performed using the GenScript Taq DNA polymerase (#E00007; GenScript). The primer sequences were as follows: forward 5′‐CTTCTGGAGTGTTTGAAGAC‐3′, reverse 5′‐CTGGAACTCACTCTGAAGAC‐3′. RNA was considered free of gDNA contamination when no product was detected by agarose gel electrophoresis.

RNA was reverse‐transcribed using the High Capacity cDNA Reverse Transcription Kit (#4368813; Thermo Fisher Scientific) in the presence of an RNase inhibitor (RNaseOUT™, #10777019; Thermo Fisher Scientific) following the manufacturer's instructions.

qPCR was performed using the 5x HOT FIREPol EvaGreen qPCR Mix and a total volume of 20 µl. The primer sequences and annealing temperatures for each transcript are detailed in Table S1. The CFX96 touch™ Real‐Time PCR detection system (Bio‐Rad Laboratories) was used to quantify gene expression. Data were analyzed either by absolute quantification, using a standard curve of the PCR product cloned into the pGEM‐T Easy vector (#A1360, Promega), or with the comparative ΔΔCt method. Housekeeping genes were chosen based on the literature, or after evaluating the expression stability of at least three candidate genes under the experimental conditions, using the geNorm, NormFinder, and BestKeeper Software tools. Absolute amounts of the transcript were normalized to the corresponding housekeeping genes.

### 4.8 Western Blotting

Protein lysates were prepared in either SB lysis buffer (50 mM Tris‐HCl, 1% SDS, 10% glycerol, 5% β‐mercaptoethanol, 0.004% bromophenol blue, in water) or RIPA buffer (50 mM Tris‐HCl, 1% Triton X‐100, 0.1% SDS, 0.5% sodium deoxycholate, 150 mM NaCl, in water), supplemented with a protease inhibitor cocktail (cOmplete^®^ Mini, #04693124001; Roche). Samples were sonicated for 5 s and stored at −80°C until further analysis. The protein concentration in RIPA lysates was measured using the Pierce™ BCA Protein Assay Kit (#23225; Thermo Fisher Scientific) (Dembek et al., 2017; Hoppstädter, Diesel, et al., 2019).

The cell fractionation was performed based on a published method (Suzuki, Bose, Leong‐Quong, Fujita, & Riabowol, 2010). Briefly, cells were washed with ice‐cold PBS once and detached by scraping. After centrifugation (4°C, 12,000 *g*, 30 s) the supernatant was discarded, and the cell pellet was resuspended in 0.1% NP‐40 in PBS. The supernatant obtained after centrifugation (4°C, 12,000 *g*, 30 s) represented the cytosolic fraction. The remaining pellet, that is, the nuclear fraction, was resuspended in 0.1% NP‐40 in PBS. One volume of 2x Laemmli buffer with β‐mercaptoethanol (100 mM Tris‐HCl, pH 6.8, 2% SDS, 20% glycerol, 10% β‐mercaptoethanol, 0.008% bromophenol blue), supplemented with a protease inhibitor cocktail (cOmplete^®^ Mini, #04693124001; Roche), was added to one volume of sample. After sonication, samples were boiled at 95°C for 1 min and were stored at −80°C until further analysis.

SDS–polyacrylamide gel electrophoresis, blotting, and staining were performed as described previously. Signals were detected using an Odyssey^®^ Near‐Infrared Imaging System and software (LI‐COR Biosciences) (Dembek et al., 2017; Hoppstädter, Dembek, et al., 2019; Hoppstädter, Diesel, et al., 2019; Hoppstädter et al., 2016).

### 4.9 Flow cytometry

Murine spleens and mesenteric lymph nodes were disrupted manually by carefully pushing them through a 40‐µm EASYstrainer (#542040; Greiner Bio‐One) while washing with RPMI 1640 without additives. Peripheral blood samples (50 µl) were collected from the tail vein and diluted in 1 ml cold PBS‐EDTA. Cells were centrifuged, resuspended in erythrocyte lysis buffer (155 mM NH_4_Cl, 10 mM KHCO_3_, 1 mM Na_2_EDTA) and incubated on ice for 10 min. Liver samples were generated by digestion of the left lateral liver lobe using the liver dissociation kit (#130‐105‐8807; Miltenyi Biotec) and the gentleMACS Octo Dissociator (Miltenyi Biotec) according to the manufacturer's instructions.

Cells were washed with PBS and stained with a viability dye (Zombie Yellow, #423101; BioLegend) at room temperature in the dark for 20 min, followed by a blocking step with mouse BD Fc Block™ (#553142) at room temperature for another 10 min. Extracellular markers were stained by incubation with an antibody cocktail diluted in Brilliant stain buffer (#563794; BD Biosciences) on ice in the dark for 30 min. The composition of the antibody cocktails is given in Table S2. Cells were washed with FACSwash (PBS with 2.5% FCS and 0.05% sodium azide) and fixed for 30 min on ice in eBioscience™ IC Fixation Buffer (1:1 in PBS, #00‐8222–49; Thermo Fisher Scientific), followed by intracellular staining with either anti‐GILZ‐PE or the corresponding isotype control.

For intracellular staining, cells were permeabilized with saponin buffer (0.2% saponin in FACSwash) for 10 min at room temperature and blocked on ice in saponin blocking buffer (PBS with 20% FCS and 0.2% saponin) for 30 min. After centrifugation, cells were resuspended in 200 µl saponin buffer. 100 µl of the cell suspension were incubated with anti‐GILZ‐PE (5 µg/ml), and 100 µl were incubated on ice and in darkness with the appropriate isotype control (5 µg/ml) for another 30 min. Subsequently, samples were washed in saponin buffer and resuspended in eBioscience™ IC Fixation Buffer. The specificity of the anti‐GILZ antibody was validated by negative staining of GILZ knockout cells (Hoppstädter et al., 2016).

Sample analysis was performed on a BD LSRFortessa (BD Biosciences) using BD FACSDiva 8.0 software. The ArC amine‐reactive compensation kit (Invitrogen, #A10346), anti‐rat/hamster beads (BD Biosciences, #552845), or anti‐mouse plus beads (BD, #560497) were used to generate compensation controls. All gates were set by using the appropriate fluorescence minus one (FMO) control (Kessler et al., 2019).

### 4.10 Determination of steroid levels via LC‐HRMS/MS

The determination of steroid levels was performed using liquid chromatography–high‐resolution mass spectrometry (LC‐HRMS/MS). Samples were mixed 1:1 with an internal standard (cortisol‐D4, 100 µg/ml in acetonitrile containing 0.1% formic acid), and centrifuged at 18,407 *g* and −10°C for 10 min. The supernatant was transferred into an MS vial, and a volume of 5 µl was used for analysis.

Chromatographic separation of the analytes was carried out on a Dionex UltiMate UHPLC System (Thermo Fisher Scientific) using an Accucore™ Phenyl‐Hexyl LC column (100 mm × 2.1 mm, 2.6 µm) heated to 40°C. Mobile phase A was water with 0.1% formic acid and ammonium formate; mobile phase B was acetonitrile with 0.1% formic acid. The LC gradient was as follows: Starting with 2% solvent B over 0.1 min, the gradient was increased to 98% solvent B until 5 min, maintained at 98% until 7.5 min, and decreased to 2% until 10 min. The flow rate was set to 600 µl/min. Detection of analytes was achieved via high‐resolution mass spectrometry on a Thermo Fisher Q‐Exactive Plus equipped with heated electrospray ionization (HESI)‐II source. The HESI‐II source conditions were as follows: sheath gas, nitrogen, at 55 arbitrary units; auxiliary gas, nitrogen, at 15 arbitrary units; temperature, 450°C; spray voltage, 3.50 kV; ion transfer capillary temperature, 275°C; and S‐lens RF level, 55.0. Mass spectrometry was done in positive polarity mode using targeted single ion monitoring (tSIM) mode. The settings for tSIM mode were as follows: resolution, 35,000; microscans, 1; AGC target, 5e4; maximum IT, 200 ms; isolation window, 2.0 m/z; normalized collision energy (NCE), 35; scan range, m/z 150–900; spectrum data type, profile; and underfill ratio, 0.5%. The peak areas of the analytes were normalized using the internal standard peak area ratio.

### 4.11 Determination of 11β‐HSD1 activity in peritoneal macrophages

The activity of 11β‐HSD1 in PMs from young and aged mice was measured after incubating the cells with 0.1 µM or 1 µM cortisone‐D8 for 1 or 24 hr. At the end of treatment, supernatants were collected and cortisone‐D8 and cortisol‐D8 levels were analyzed as described under 1.9. The conversion to cortisol‐D8 was expressed as a percentage of the total steroid amount measured and is presented as a ratio to total cellular protein content within the well. Protein concentrations were determined after lysing the cells in RIPA buffer by using the Pierce™ BCA Protein Assay Kit (#23225; Thermo Fisher Scientific) as recommended by the supplier.

### 4.12 GTEx data retrieval and expression analysis

The data used for the analyses described in this manuscript were downloaded from the GTEx Portal on 02/14/20, corresponding to GTEx Analysis data V8 (GTEx Consortium, 2017). Expression data (TPM values) in the file GTEx_Analysis_2017‐06‐05_v8_RNASeQCv1.1.9_gene_tpm.gct were linked to meta‐information using GTEx_Analysis_v8_Annotations_SampleAttributesDS.xls and GTEx_Analysis_v8_Annotations_SubjectPhenotypesDS.txt files in the R programming language.

### 4.13 Statistics

Box plots show the 25–75th percentiles (box), mean (circle), median (line), and *SD* (whiskers). Results within bar graphs are expressed as mean ± *SEM*. Statistically significant differences between means were determined using the GraphPad Prism 6.0 or Origin 2019 software. Outliers were determined using the Grubbs’ test or Dixon's *Q* test. Unless stated otherwise, an unpaired Welch's *t* test was performed for the comparison of two groups, and the comparison of three or more groups was carried out by one‐ or two‐way analysis of variance (ANOVA) followed by Bonferroni's post hoc analysis for individual differences. Where specified, median comparison of two groups was performed using Mann–Whitney *U* test. Results were considered significant at *p* < .05.

## CONFLICT OF INTEREST

The authors declare no conflict of interest.

## AUTHOR CONTRIBUTIONS

JVVP, RL, AD, MHS, MRM, and JH conducted experiments and acquired and analyzed the data. JVVP and JH wrote the manuscript. SB, CR, MRM, AKK, and JH supervised and designed research studies and revised the manuscript.

## Supporting information

Supplementary Material

## Data Availability

The data used for the analyses described in this manuscript were downloaded from the GTEx Portal on 02/14/20, corresponding to GTEx Analysis data V8 (GTEx Consortium et al., 2017).
