# Supplementary material for: Altered glucocorticoid metabolism represents a feature of macroph‐aging
Source: Aging Cell. 2020 May 28;19(6):e13156. doi: 10.1111/acel.13156 (PMC7294787; doi:10.1111/acel.13156)
Supplement: Supplementary file 1 — Supplementary Material [file ACEL-19-e13156-s001.pdf]

Supporting information

Altered glucocorticoid metabolism represents a feature of macroph-aging

Jenny Vanessa Valbuena Perez[1*], Rebecca Linnenberger[1*], Anna Dembek[1], Stefano Bruscoli[2], Carlo Riccardi[2], Marcel H. Schulz[3], Markus R. Meyer[4], Alexandra K. Kiemer[1], Jessica Hoppstädter[1#]

[1]Pharmaceutical Biology, Department of Pharmacy, Saarland University, Saarbrücken, Germany

[2]Pharmacology, Department of Medicine, Perugia University, Perugia, Italy

[3]Institute for Cardiovascular Regeneration, Goethe University Hospital, Frankfurt, Germany

[4]Department of Experimental and Clinical Toxicology, Institute of Experimental and Clinical Pharmacology and Toxicology, Center for Molecular Signaling (PZMS), Saarland University, Homburg, Germany

*Equal contribution.

[#]Correspondence:

Jessica Hoppstädter, Ph.D.
Pharmaceutical Biology
Department of Pharmacy
Saarland University
66123 Saarbrücken, Germany
Phone: +49 681 302 57304
Fax: +49 681 302 57302
Email: j.hoppstaedter@mx.uni-saarland.de

1. Supplementary figures

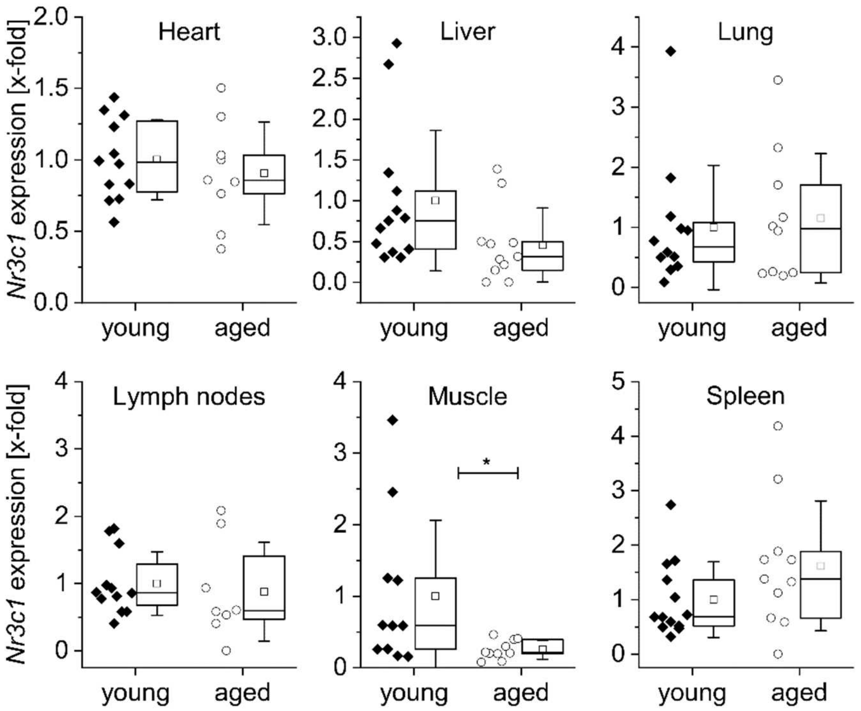

Supplementary figure S1. *GR expression across tissues*. GR (*Nr3c1*) expression in tissues from young and aged mice. mRNA expression levels were measured in heart, liver, lung, lymph nodes, skeletal muscle, and spleen tissues, normalized against the housekeeping gene *Ppia*, and expressed as x-fold of young (n=9-13). Box plots show the 25–75th percentiles (box), mean (square), median (line), and SD (whiskers). *$p<0.05$ relative to young mice, as determined by two-tailed t-test.

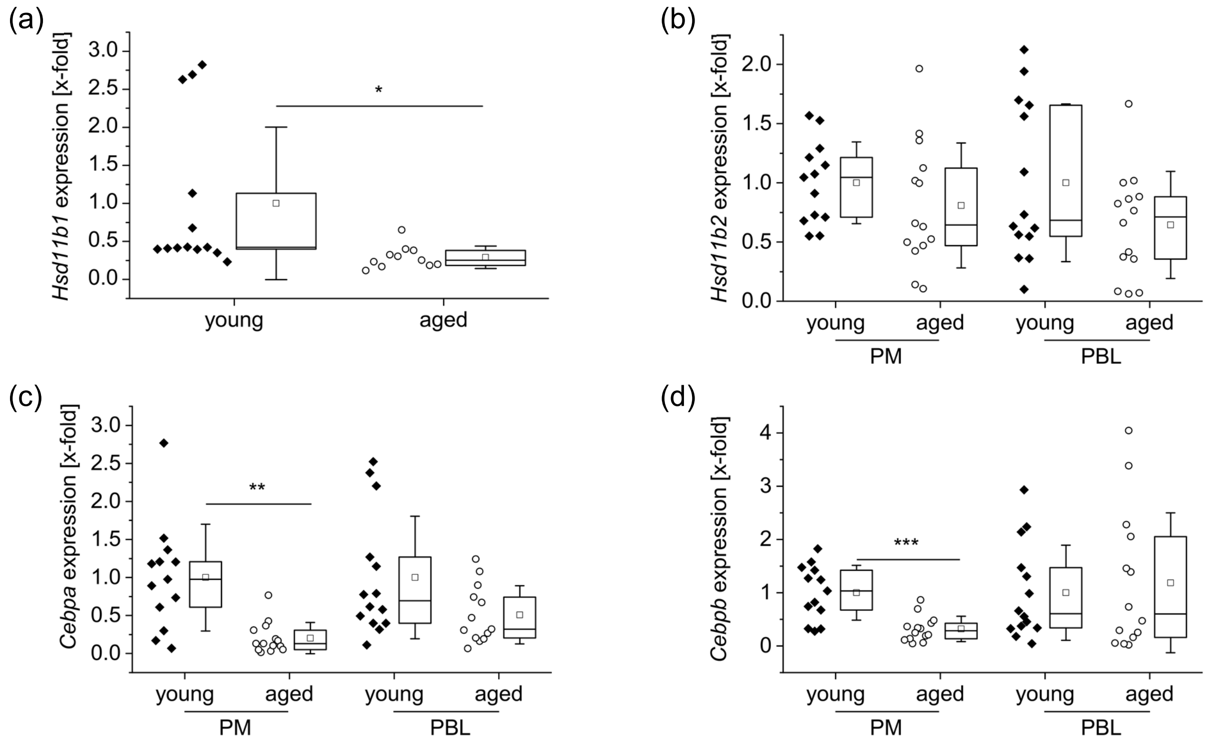

Supplementary figure S2. *Alterations in the expression of genes involved in GC metabolism.* (a) *Hsd11b1* expression in livers of young and aged mice (young: n=13, aged: n=11) was measured by qPCR, normalized to *Ppia*, and expressed as x-fold of young. (b-d): *Hsd11b2* (b), *Cebpa* (c), and *Cebpb* (d) expression levels were measured in PMs and PBLs (n=14), normalized to *Ppia*, and expressed as x-fold of young. Box plots show the 25–75th percentiles (box), mean (square), median (line), and SD (whiskers). *p<0.05, **p<0.01, ***p<0.001 by two-tailed t-test.

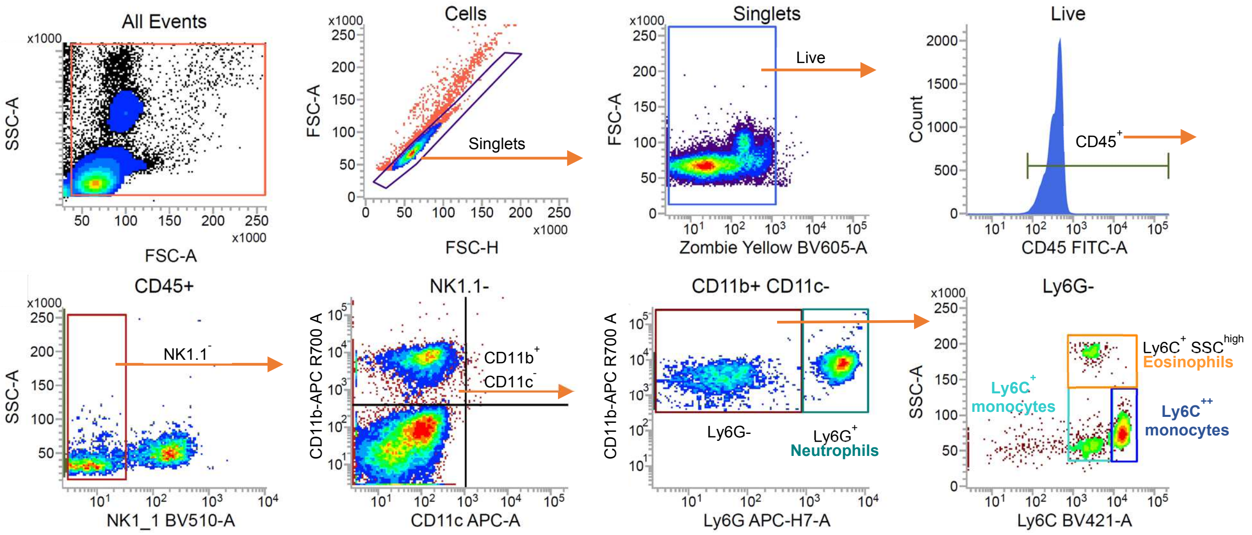

Supplementary figure S3. *Determination of GILZ expression in myeloid cells in peripheral blood from young and aged mice.* A: Gating strategy for the quantification of GILZ in myeloid cells from tail vein blood. Gates were set using fluorescence minus one (FMO) controls (not shown).

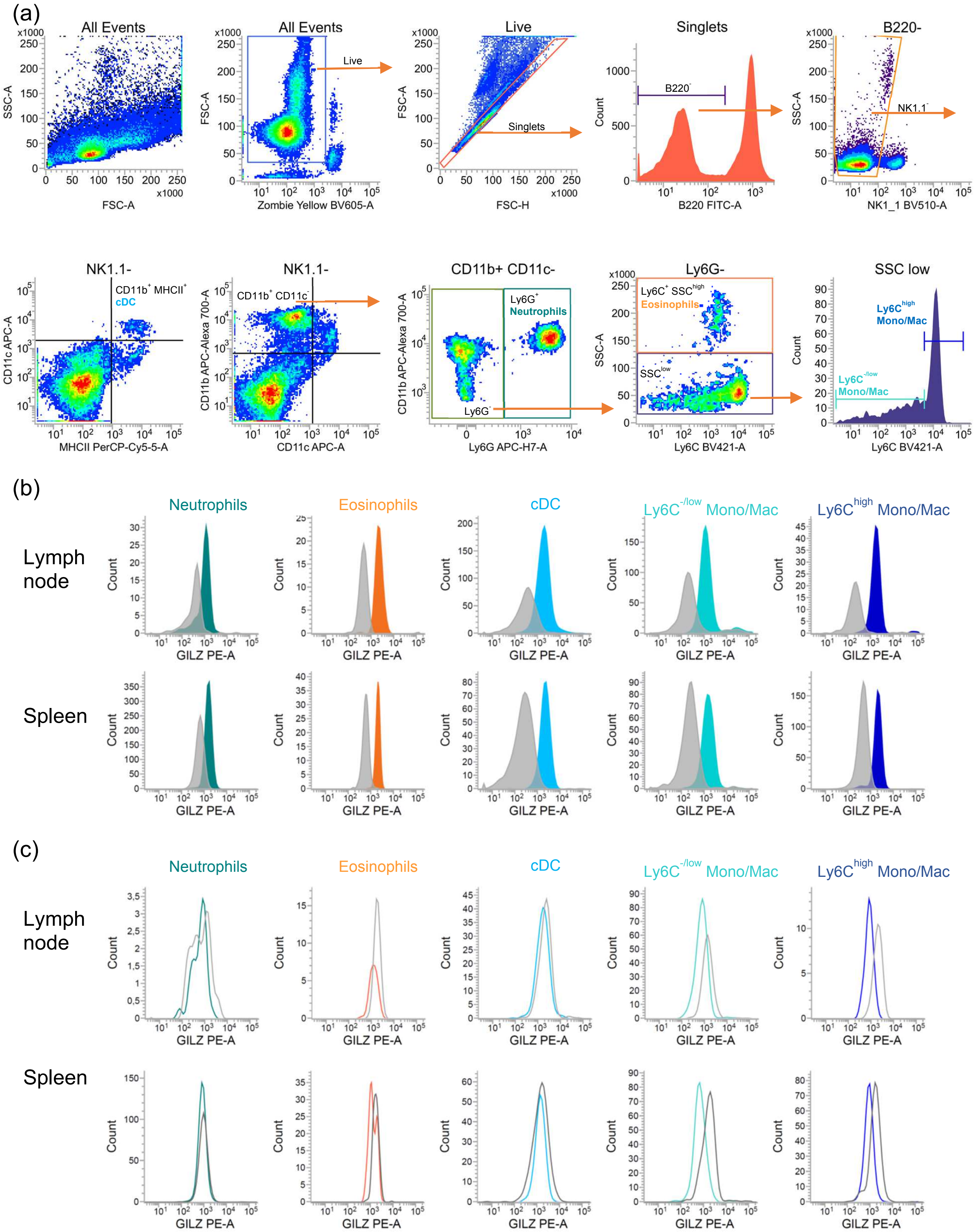

Supplementary figure S4. *Determination of GILZ expression in myeloid cells in lymphoid tissues from young and aged mice.* (a) Gating strategy for the quantification of GILZ in myeloid cells from lymphoid tissues. Gates were set using fluorescence minus one (FMO) controls (not shown). (b) Representative histograms showing GILZ expression in myeloid subsets in young mice. Colored: GILZ signal, gray: isotype control. (c) Representative histograms showing GILZ signals in cells from aged and young mice. Gray line: young; colored line: aged.

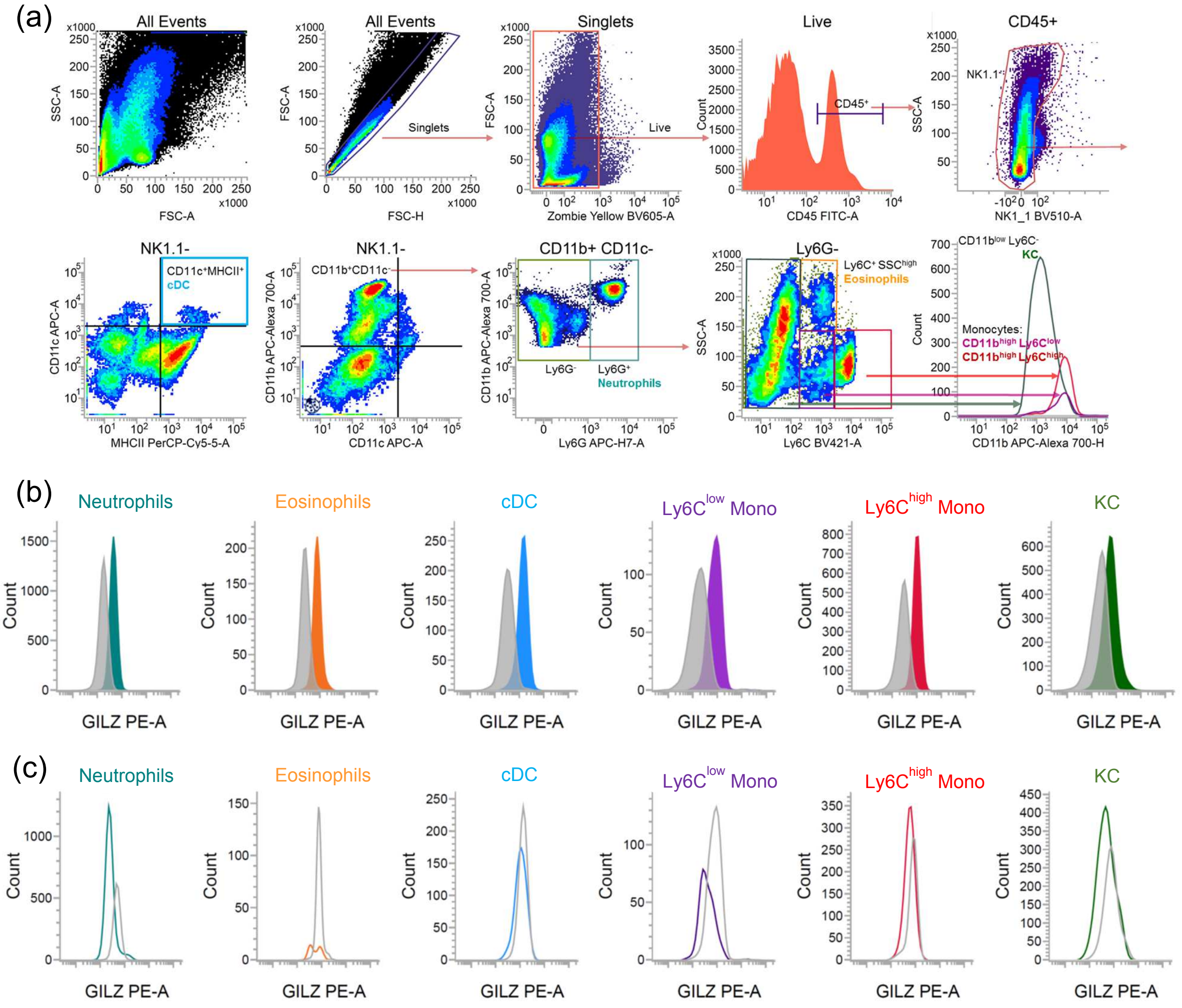

Supplementary figure S5. *Determination of GILZ expression in myeloid cells in liver tissue from young and aged mice.* (a) Gating strategy for the quantification of GILZ in myeloid cells from liver tissue. Gates were set using fluorescence minus one (FMO) controls (not shown). (b) Representative histograms showing GILZ expression in myeloid subsets in young mice. Coloured: GILZ signal, gray: isotype control. (c) Representative histograms showing GILZ signals in cells from aged and young mice. Gray line: young; colored line: aged.

2. **Supplementary tables**

Supplementary table S1: qPCR conditions.

Gene	NCBI Accession number	Forward primer sequence 5´-3´	Reverse primer sequence 5´-3´	µl primer [10 µM] / 20 µl reaction	Annealing T (°C)
Human					
ACTB	NM_001101.3	TGCGTGACATTAAGG AGAAG	GTCAGGCAGCTCGTA GCTCT	0.5	60
ANXA1	NM_000700.3	CTCACAGCTATCGTGA AGTGC	TGCCTTATGGCGAGTT CCAA	0.5	60
DUSP1	NM_004417.4	CAGCTGCTGCAGTTT GAGTC	AGGTAGCTCAGCGCA CTGTT	0.5	60
SGK1	NM_005627.4	GGACTCTGCAAGGAG AACATTG	GCTGCTTATGAAGCAC CTCAG	0.5	60
TSC22D3 (GILZ)	NM_004089.3	TCCTGTCTGAGCCCTG AAGAG	AGCCACTTACACCGCA GAAC	0.5	60
Mouse					
Abca1	NM_013454.3	ACAAGTCCATCGTGTC TCGC	GGGATGCTTGATCTGC CGTA	0.5	60
Abcg1	NM_009593.2	ACACCGATGTGAACC CGTTT	CAGATGTGTCAGGAC CGAGT	0.5	60
Apoe	NM_009696.3	CAGTGGCCCAGGAGA ATCAAT	TCACAGAGACTCAGA ATGTGC	0.5	60
Cdkn1a	NM_007669.5	GACCAGCCTGACAGA TTTCTA	TGGGCACTTCAGGGTT TTCT	0.5	60
Cdkn2a	NM_009877.2	CGGGGACATCAAGAC ATCGT	GCCGGATTTAGCTCTG CTCT	0.5	60
Cebpa	NM_0012875 23.1	TTCGGGTCGCTGGATC TCTA	TCAAGGAGAAACCAC CACGG	0.5	60
Cebpb	NM_0012877 39.1	GGAGACGCAGCACAA GGT	AGCTGCTTGAACAAG TTCCG	0.5	60
Csnk2a2	NM_009974.3	GTAAAGGACCCTGTGT CAAAGA	GTCAGGATCTGGTAG AGTTGCT	0.8	60
Cyp11a2	NM_019779.4	ATGAGATCCCTTCCCC TGGC	TGCCCAGCTTCTCCCT GTAAA	0.5	60
Hmgcr	NM_008255.2	ATCCAGGAGCGAACC AAGAGAG	CAGAAGCCCCAAGCA CAAAC	0,5	60
Hsd11b1	NM_008288.2	GGAACCCAGGAAGGA AGATCA	CAGGCAGGACTGTTCT AAGAC	0.5	60
Hsd11b2	NM_008289.2	AACCTCTGGGAGAAA CGCAAG	GGCATCTACAACTGG GCTAAGG	0.5	60
Il1b	NM_008361.3	CCAAAAGATGAAGGG CTGCTT	GGAAGGTCCACGGGA AAGAC	0.5	60
Il6	NM_031168.2	AAGAAATGATGGATG CTACCAAACTG	GTACTCCAGAAGACC AGAGGAAATT	0.4	60

Ldlr	NM_010700.3	TCAATGGGGGCAATC GGAAA	ACACTTTGTCCTCATA GATGGC	0.5	60
Nr3c1	NM_008173.3	AAAGAGCTAGGAAAA GCCATTGTC	TCAGCTAACATCTCTG GGAATTCA	0.5	61
Ppia	NM_008907.1	GGCCGATGACGAGCC C	TGTCTTTGGAACTTTG TCTGC	0.5	58
Scarb1	NM_016741.2	TCCTGAAGACACTATA AGCCCC	GTGCGGACAGGTGTG ACAT	0.5	60
Sirt1	NM_019812.3	TGGAGCAGGTTGCAG GAATC	GGCACCGAGGAACTA CCTGAT	0.5	60
Stard1	NM_011485.5	TGTACCAAGCGCAGA GGTTC	GGCCGTGTTCAGCTCT GATG	0.5	60
Tnf	NM_013693.2	CCATTCCTGAGTTCTG CAAAGG	AGGTAGGAAGGCCTG AGATCTTATC	0.5	60
Tsc22d3 (Gilz)	NM_010286.4	GCTGCTTGAGAAGAA CTCCCA	GAACTTTTCCAGTTGC TCGGG	0.5	60

Supplementary table S2: Antibody cocktails. All antibodies were used at a final concentration of 5 µg/ml.

**Extracellular staining cocktail**	
Peripheral blood / Liver	***Lymphoid tissues***
CD45-FITC	CD45R/B220-FITC
NK-1.1Brilliant Violet™ 510	NK-1.1Brilliant Violet™ 510
CD11c-APC	CD11c-APC
CD11b-APC-R700	CD11b-APC-R700
I-A/I-EPerCP-Cy™5.5	I-A/I-EPerCP-Cy™5.5
Ly-6G-APC-H7	Ly-6G-APC-H7
Ly-6C-Brilliant Violet™ 421	Ly-6C-Brilliant Violet™ 421
